# Comparison of different weight-based scalars of remimazolam tosylate for anesthesia induction in obese patients: study protocol for a prospective, controlled trial

**DOI:** 10.1186/s13063-023-07739-6

**Published:** 2023-11-10

**Authors:** Wenwen Ni, Xiuwen Yi, Lili Feng, Yilei Shen, Jiali Jiao, Yirong Cai, Danyun Fu, Yuan Han, Ji’e Jia, Wenxian Li

**Affiliations:** 1 Department of Anesthesiology, Eye & ENT Hospital, Fudan University, No. 83 Fenyang Road, Xuhui District, Shanghai, 200031 China; 2 Institute of Translational Medicine, Shanghai Jiao Tong University, 800 Dongchuan Road, Minhang District, Shanghai, China

**Keywords:** Obese, Remimazolam tosylate, Induction dose, Lean body weight, Randomized controlled trial

## Abstract

**Background:**

The physiologic and anthropometric characteristics changes associated with obesity may result in the alternation of pharmacologic management. Remimazolam tosylate is a new type of ultra-short-acting benzodiazepine with stable context-sensitive half-time (CSHT) and no lipid accumulation after long-time infusion. Although remimazolam tosylate has potential advantages for the induction and maintenance of anesthesia in obese patients, the appropriate induction dosing scalars among obese patients are unknown. Therefore, we aim to compare the different weight-based scalars for dosing remimazolam tosylate of anesthesia induction among obese patients.

**Methods/design:**

The study will be performed as a prospective, single-center, double-blind, controlled clinical trial. The study design is a comparison of remimazolam tosylate requirements based on total body weight (TBW) or lean body weight (LBW) to reach a Modified Observer’s Assessment of Alertness and Sedation (MOAA/S) score of 0 among obese subjects (BMI ≥ 35 kg/m^2^). Another twenty normal-weight subjects (18.5 kg/m^2^ ≤ BMI < 25 kg/m^2^) will be enrolled as a control group, whose induction dose is scaled based on TBW. The infusion rate of remimazolam tosylate during induction is 12 mg/kg/h in all groups.

**Discussion:**

Results of the present study will provide evidence of dose scalar of remimazolam tosylate to guide the clinical practice of anesthesia induction in obese patients.

**Trial registration:**

Chinese Clinical Trial Registry ChiCTR220005664. Registered on 9 February 2022, https://www.chictr.org.cn/showproj.aspx?proj=151150.

## Background

Obesity is a recognized global health problem [1]. Physiological changes related to obesity alter the pharmacokinetics (PK) and pharmacodynamics (PD) and narrow the therapeutic window of anesthetics [2, 3]. Thus, it is necessary to consider the proper dose of anesthetic drugs among obese patients, particularly morbidly obese (MO) patients.

Remimazolam is a new ultra-short-acting benzodiazepines [4], which shows positive modulation on the four major subtypes (α1, α2, α3, α5) of the GABA_A_ receptor [5, 6] and is rapidly metabolized by tissue esterase to an inactive carboxylic acid metabolite [7]. The context-sensitive half-time (CSHT) of it hardly changes even after long-term infusion [8–10]. Besides, there is a specific antagonist for remimazolam in clinical settings [11]. All of the above reasons make remimazolam a potentially suitable sedation and induction anesthetic in obese patients [12, 13]. Among normal-weight patients, remimazolam is recommended to be administered according to TBW.

However, among obese patients, many factors, such as the lipophilicity of the drug, ionization properties, blood to plasma ratio, and protein binding, influence the volume of distribution (Vd) [14]. Vd is of particular significance for choosing the optimal loading dose, but TBW is not always a strongly linear increase with the Vd of a drug [14, 15]. Lean body weight (LBW) is TBW minus the weight of the fat mass, which has a higher correlation with cardiac output (CO) than the fat mass, thus making it ideal for determining loading and induction doses among obese patients [16]. In general, there is no single weight-based scalar that is undeniably better than the others for describing the PK of drugs in obese patients, who are often excluded from clinical trials, leading to a lack of evidence-based dosing recommendations for many drugs, especially new drugs [17]. Therefore, as a new drug, the dose scalar of remimazolam tosylate (based on TBW or LBW) among obese patients needs to be explored.

### Objectives

The primary trial objective is to determine an appropriate scalar (TBW or LBW) of remimazolam tosylate for anesthesia induction in obese patients by measuring the total induction dose (mg/kg) at the time of loss of consciousness (LOC).

## Methods

### Trial design and study setting

The exploratory study is designed as a prospective, double-blind controlled trial, which is conducted at the Department of Anesthesiology, Eye & ENT Hospital, Fudan University in Shanghai. It is registered in the Chinese Clinical Trial Registry (ChiCTR2200056641, Date of registration: February 9, 2022). The protocol is reported following the SPIRIT reporting guidelines [18].

### Participants

#### Eligibility criteria

**Fig. 1:**
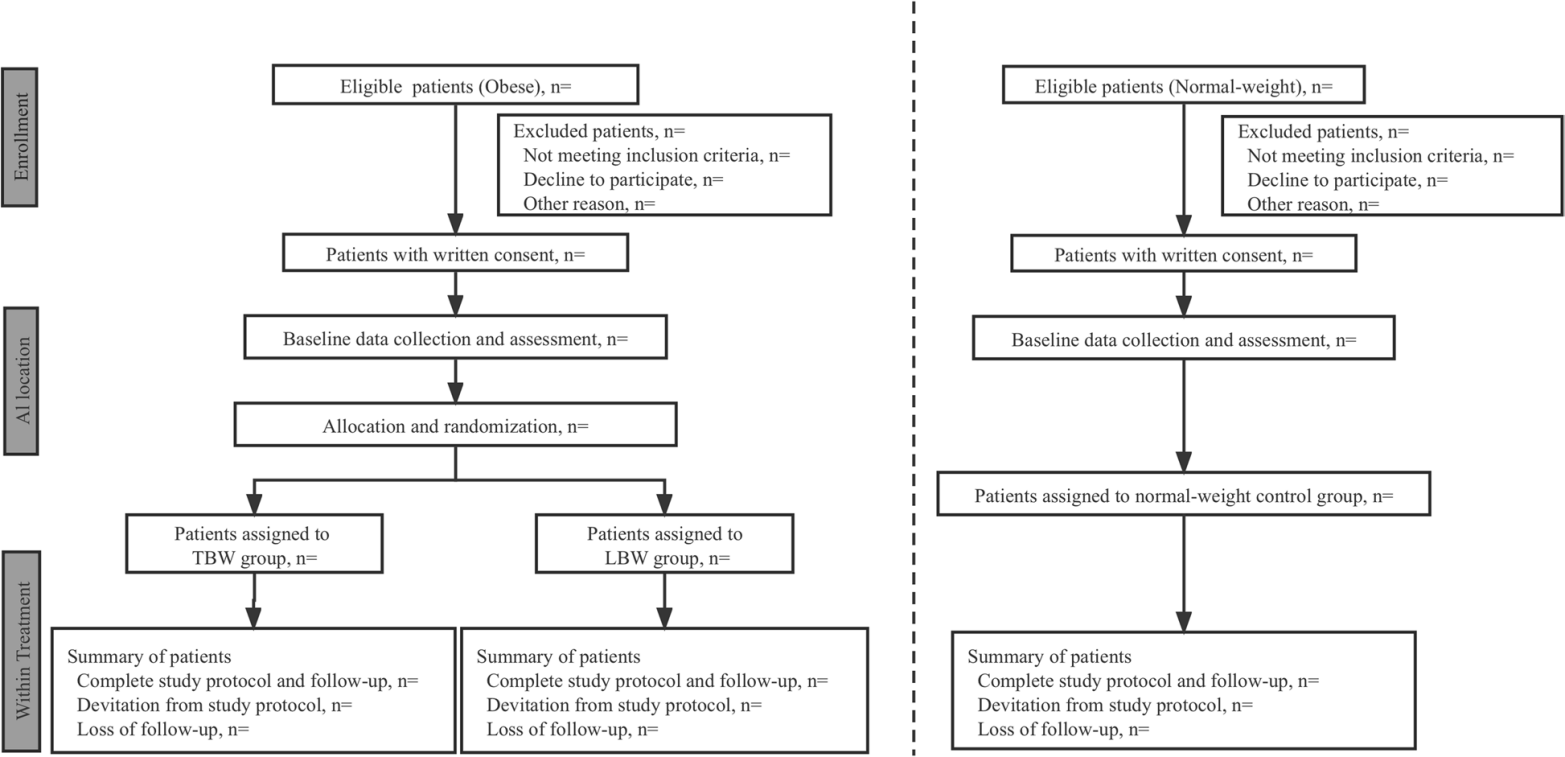
Flow chart

Forty obese patients (18 ≤ age ≤ 65 years, American Society of Anesthesiologists (ASA) grades II to III, BMI ≥ 35 kg/m^2^) are randomized to either TBW or LBW group, while twenty normal-weight control group patients (18 ≤ age ≤ 65 years, ASA I to III, 18.5 kg/m^2^ ≤ BMI < 25 kg/m^2^) receive induction dose based on TBW (Fig. 1), who are scheduled to undergo elective eye, ear, nose, throat surgery under general anesthesia and will be enrolled in this clinical trial.

#### Exclusion criteria

Patients who meet any of the following criteria will be excluded:

1. Allergic reaction to any compositions of the planned medication
2. Long-term use of analgesics, benzodiazepines, and any prescribed or over-the-counter sleep medications
3. History of significant cardiac, pulmonary, liver, or renal disease
4. History of mental abnormalities
5. Long-term drinking history
6. Participated in clinical trials of drugs as subjects in recent 3 months
7. History of difficult tracheal intubation, or an anticipated difficult airway scheduled for awake fiberoptic intubation

**Fig. 2:**
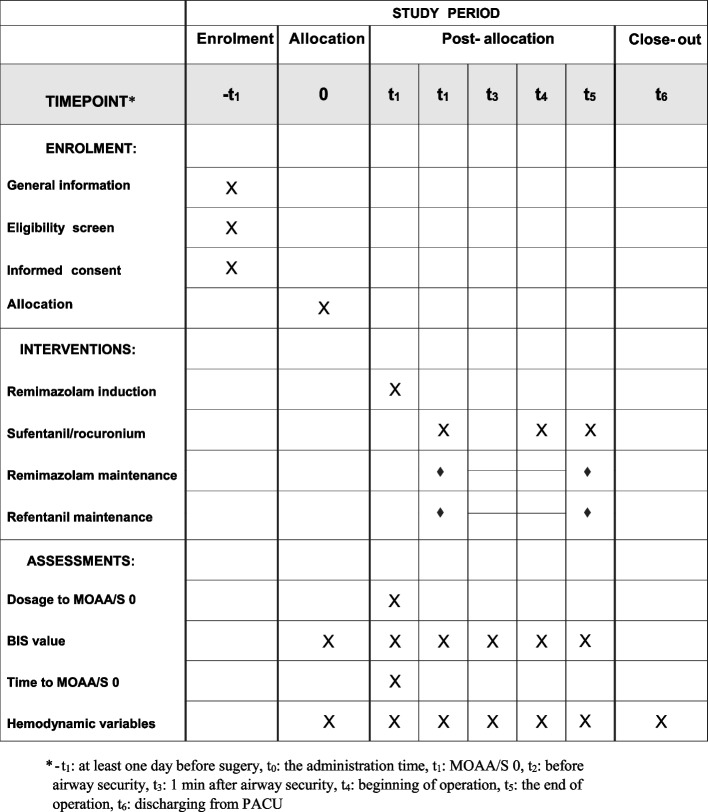
SPIRIT timeline of this trial

The schedule of enrollment, intervention, and assessment is reported according to the SPIRIT statement (Fig. 2).

The number of excluded patients and the reasons for their exclusion will be reported according to the SPIRIT statement.

#### Consent

At least 1 day before the day of surgery, the researchers will screen the potential participants from the list of scheduled operations according to the inclusion and exclusion criteria. Patients who meet the inclusion/exclusion criteria will be given written informed consent and preoperative evaluation.

The principal investigator or physician who examines the subject may decide to remove the subject from the study if the subject has an emergency medical problem (allergic reaction or acute health problem).

### Ethical approval

The study protocol (V1.0, issue date January 2022) has been approved by the Ethics Committee of Eye & ENT Hospital, Fudan University in Shanghai (2022012).

### Assignment of interventions: randomization and allocation

Forty obese patients are randomly allocated in a 1:1 ratio to either the TBW group or the LBW group after they sign the informed consent form. An independent statistician who was not directly involved with the study randomly allocated the obese patients into the TBW or LBW group according to a computer-generated randomization scheme. Patient assignments will be placed in sequentially numbered, opaque, sealed envelopes. The randomization scheme is kept inaccessible throughout the study period.

After the obese subject enters the operation room, the research nurse, who does not participate in anesthesia, will open the sealed envelope for random numbers, calculate, and set the infusion speed according to the group information.

### Assignment of interventions: blinding and procedure for unblinding if needed

Considering that the attending anesthesiologist and anesthesia assistant will be responsible for observing the clinical manifestations, including scoring of MOAA/S, they should be blinded to the allocation of the obese groups. The subjects of obese groups are blinded to the allocation. In the events of serious adverse events (SAEs) such as a medical emergency, which require identifying the intervention administered to the patient, the attending anesthesiologist or anesthesia assistant is permitted to inquire with the unblinded research nurse. Due to the induction dose based on TBW in the control group, it is difficult to double-blind.

### Intervention

#### Preparation before general anesthesia

Benzodiazepines as premedication in the study are not allowed to be provided. Other kinds of anti-anxious drugs, such as clonidine, are allowed to be provided on the night before surgery. After routinely fasting before the operation, we will insert an 18- or 20-gauge peripheral IV catheter into the subject’s left or right forearm and continuously infuse normal saline of 5 ml/kg prior to the induction of general anesthesia. All obese subjects are positioned in the “ramped” position (elevate the patients’ upper body and head) to achieve horizontal alignment between the external auditory meatus and sternal notch [19]. Heart rate (HR), noninvasive blood pressure (BP), electrocardiogram (ECG), end-tidal carbon dioxide (ETCO_2_), and pulse oxygen saturation (SpO_2_) are routinely monitored (Mindray BeneView T8, Shenzhen Mindray Bio-Medical Electronics Co) during the induction and maintenance of general anesthesia. BIS sensor (BIS™XP, Medtronic) will be applied to monitor the depth of anesthesia and the BIS value will be recorded. The acceleromyography (TOF-watch SX; MSD) will be used to monitor muscle relaxation. Keep the operation room quiet, and the patients are asked to keep their eyes closed during induction.

#### Induction of general anesthesia

After routine monitoring, preoxygenation will be given with a mask by using 100% oxygen at a flow rate 8 L/min; the anesthesia assistant will ask the patients to deep breathe continuously. The administration of the study drug will begin when the end-tidal concentration of oxygen is stable above 90% [20]. The administration time is defined as the beginning of anesthetic induction (t_0_).

For obese subjects, the remimazolam tosylate (36 mg, prepared as a 2 mg/ml solution with normal saline) will be infused at the infusion rate of 12 mg/kg/h based on TBW (*n* = 20) or LBW (*n* = 20). LBW is calculated using the Janmahasatian equation [21].

$$\begin{array}{l}\mathrm{LBW\, }(\mathrm{kg})\mathrm{\, in\, men }= \frac{9270\cdot TBW}{6680+(216\cdot BMI)}\\ \mathrm{LBW\, }(\mathrm{kg})\mathrm{ \,in\, women }= \frac{(9270\cdot TBW)}{8780+(244\cdot BMI)}\end{array}$$

For normal-weight control group (*n* = 20), remimazolam tosylate (2 mg/ml) will be infused at the infusion rate of 12 mg/kg/h based on TBW. Thirty seconds after administration, the investigator will assess the change in the level of consciousness every 10 s using the responsiveness scores of the Modified Observer’s Assessment of Alertness/Sedation Scale (MOAA/S) (Table 1) [22]. When the subject gives a negative response on opening his or her eyes and moving his or her toes, then he or she will be given a painful trapezius squeeze. Loss of consciousness (LOC) is confirmed when the first MOAA/S score reaches zero (i.e., the patient does not respond to a painful trapezius squeeze). The investigator will record the time required for LOC, and the value of BIS, HR, and BP. At MOAA/S of zero, the induction dose will be stopped, and the maintenance dose of remimazolam tosylate from 1 mg/kg/h (calculated by TBW, up to 3 mg/kg/h) will be continued. After LOC, all MO subjects are given rocuronium (0.6 mg/kg, IV. IBW) and sufentanil (0.2 μg/kg, IV. LBW). Visible arm movement during rocuronium administration is defined as rocuronium injection pain. IBW is calculated using the following formula.

**Table 1:** Modified Observer’s Assessment of Alertness/Sedation

Responsiveness	Score
Responds rapidly to name spoken in normal tone	5 (alert)
Lethargic response to name spoken in normal tone	4
Responds only after name is called loudly and/or repeatedly	3
Responds only after mild prodding or shaking	2
Responds only after painful trapezius squeeze	1
Does not respond to painful trapezius squeeze	0

$$\begin{array}{l}\mathrm{IBW\, in\, men }=\mathrm{ height }\left(\mathrm{cm}\right)-105\\ \mathrm{IBW\, in\, women }=\mathrm{ height }\left(\mathrm{cm}\right)-110\end{array}$$

All patients will be mask-ventilated after LOC, and the SpO_2_ during induction (from the administration of remimazolam tosylate to 5 min after securing the airway) will be recorded. If necessary, the oropharyngeal airway will be used for the maintenance of oxygenation. When the train-of-four (TOF) value is 0, the endotracheal intubation or supraglottic airway devices (SADs) will be placed. Then, pressure-controlled ventilation with a breathing frequency of 10–12 times/min, a ratio of inspiratory to expiratory of 1:1.5–2, and a tidal volume of 8–10 mL/kg (IBW) will be performed. The respiratory parameters will be adjusted to maintain the ETCO_2_ between 35 and 45 mmHg.

#### Maintenance of general anesthesia

After LOC, the infusion rate in both groups will be adapted for maintaining the depth of anesthesia starting at a dose of 1 mg/kg/h (calculated by TBW, upper limit: 3 mg/kg/h) based on clinical signs and symptoms, such as changes in BP, HR, and keep BIS value between 40 and 60. If EMG or electrical signal interference is eliminated, the BIS value is above 60 for more than 3 min, the infusion rate of remimazolam tosylate will be increased by 0.2 mg/kg/h each time. The dose of anesthetic drugs during general anesthesia, such as remifentanil and rocuronium, will be recorded.

#### Recovery from general anesthesia

All patients will be transferred to the post-anesthesia care unit (PACU) after the surgery; they will be positioned head-up. The sugammadex (2 mg/kg, IV TBW) and flumazenil (0.5 mg per patient) are given and the patients will be sent back to the ward according to the clinical protocol and the PACU physicians’ judgments.

### Outcomes

#### Definition of the primary outcome

The primary outcome of the study is the calculated dose of per kilogram remimazolam tosylate requirement from the time point of remimazolam tosylate starting infusion to the time point of the MOAA/S score reaching zero (LOC).

#### Assessment of primary outcome

The dose requirement of remimazolam tosylate during the induction period will be calculated according to the dose per kilogram TBW in the TBW group or LBM in the LBW group. The dose requirement of remimazolam tosylate of the control group during the induction period will be calculated based on TBW.

#### Definition of secondary outcomes

The secondary outcomes include the time from the start of remimazolam infusion to MOAA/S zero, total induction dose of remimazolam tosylate when MOAA/S reaches zero, the BIS value and hemodynamic variables (HR, BP) at the baseline, MOAA/S zero and 1 min after intubation, adverse events during induction (such as injection pain, hiccup, bucking…), the dose of remifentanil, the BIS value during maintenance, and the total time stay in PACU.

### Safety management and adverse event

Researchers will make sure that the adverse event response team is on standby throughout the process. An adverse event refers to any untoward medical occurrence which happens during the trial. Adverse events include but are not limited to respiratory depression and cardiocirculatory instability. All adverse events will be treated immediately. For instance, if spontaneous respiration is insufficient during induction in obese subjects (SpO_2_ < 92%), auxiliary mask ventilation with an oropharyngeal airway will be given to the patients. Cardiovascular events should be handled promptly, with 6–12 mg ephedrine given if the patient’s systolic blood pressure is below 80 mmHg and 5 mg atropine given if the patient’s heart rate is below 45 bpm. The type of adverse event, likely cause, and treatment will be documented and discussed in data monitoring committee (DMC) meetings. If an adverse event occurs, the research team will afford free medical treatment and necessary follow-up.

### Data collection

Before surgery, general information (age, gender, height, TBW, BMI, LBW, IBW, ASA status, Mallampati score) and the surgical methods of the patients are investigated, and indexes such as albumin, alanine aminotransferase (ALT), and aspartate aminotransferase (AST) are recorded. Baseline BIS, HR, SBP, DBP, and MAP are recorded before induction (-t_1_). During induction, BIS and the hemodynamic profiles are recorded at the following time points: MOAA/S 0 (t_1_), before airway security (t_2_), 1 min after airway security (t_3_), beginning of operation (t_4_), the end of operation (t_5_), discharging from PACU (t_6_). The minimum SpO_2_ during induction (from administration to 5 min after intubation) will be recorded. Time from the start of administration of the remimazolam tosylate to MOAA/S 0, time from the end of administration to extubation, and time from the end of administration to discharge from the PACU are also recorded.

### Data management

Before the initiation of the study, electronic case report forms (CRFs) will be established with password-protected access. Each enrolled patient will be assigned an identification number. Original data will be collected on the case report forms (CRFs) by an anesthesiologist assistant blinded to the group allocation. We will indicate a coordinator to ensure the integrity of data collection and timely completion of the CRFs.

Two researchers will independently enter the data from CRFs into the database, which will be created using Microsoft Excel 2019 (Microsoft, USA). Consistency between the two entries will be tested. Patient data will be coded and kept confidential.

### Trial management and data monitoring

The compositions of the trial management group (TMG) include the principal investigators, research members, and the trial statistician team. The members of TMG will meet monthly to ensure that the progress of the study is conducted according to protocol, planned timelines, and budget. A semi-annual audit will be conducted by an independent clinical research expert from Clinical Research Unit of Eye & ENT Hospital of Fudan University, to ensure that the protocol is followed, there is no issue with the informed consent procedure, and record keeping is accurate.

The quality of data will be monitored quarterly by the data monitoring committee (DMC), which is composed of statisticians and representatives from the Ethics Committee and Clinical Research Unit of Eye & ENT Hospital, Fudan University. The members of the DMC are independent of the sponsors. After the data collection is completed, the researchers will be granted full access to the dataset.

### Participant timeline

The participant timeline is shown in Fig. 2.

### Sample size calculation

Although there was no previous study that demonstrated the optimum induction dose of remimazolam tosylate in obese patients. The sample size calculation is based on the ability to detect a difference of approximately 0.1 mg/kg between groups in the dose per kilogram of remimazolam tosylate. This is based on the studies which describe a clinically important difference of 0.12 with a standard deviation of 0.08 of the mean cumulative doses of remimazolam induction to reach LOC between 6 mg/kg/h and 12 mg/kg/h groups [23]. Based on these parameters, we calculated a sample size of 20 subjects per group with a power of 90%, alpha 0.025, and a 20% dropout rate.

### Statistical analysis

#### Statistical analysis for primary and secondary outcomes

Baseline clinical and demographic characteristics will be summarized and reported using descriptive statistics. Categorical variables will be reported as numbers and percentages and will be compared using the chi-square test or Fisher’s exact test, as appropriate. The Shapiro-Wilks test will be used to first verify the normality of the continuous variables and will be reported as means and standard deviations or as medians and interquartile ranges. Continuous variables will be compared using Student’s *t* test or the Mann-Whitney *U* test, as appropriate.

The primary analysis will examine the difference in primary outcome between groups and will be reported as an absolute difference; an unpaired two-tailed t test will be used to test the statistical significance of the mean difference between the three groups. Then, the primary outcome will be analyzed using a linear mixed model adjusted by a set of baseline variables (gender or age) that are strongly believed to affect the outcome. Secondary outcomes will be analyzed using linear, logistic, or ordered regression analysis methods as appropriate, with the same adjustments as the primary outcomes. The primary analysis will be conducted according to the intention-to-treat principle, and per-protocol analysis will be performed and reported separately. All estimated treatment effects will be accompanied by 95% confidence intervals and p-values. Analysis will be performed by use of SPSS statistical package (IBM, version 21.0; SPSS).

#### Methods in analysis to handle protocol non-adherence and any statistical methods to handle missing data

We anticipate no missing data on primary outcome due to the use of video recording by the research nurse during the induction process. However, if missing data occurs, we will take a sensitivity analysis by using the maximum bias strategy.

#### Plans for communicating important protocol amendments to relevant participants

If necessary, amendments to the protocol are to be made after the discussion between the principal investigator and other researchers. We will first notify the sponsor and funder. Then, the principal investigator will notify the center and a copy of the approval revised protocol will be sent to the Investigator Site File. In addition, the principal investigator of this study will update the revised protocol in the Clinical Trial registry. Any deviations from the protocol will be fully documented using a breach report form.

## Discussion

As an improved understanding of the physiological changes associated with obesity and its rising prevalence worldwide, the clinician has yet to recognize obese patients as a separate entity with characteristics that differ from the rest of the population. The “one size fits all” concept of dosing is gradually being replaced by strategies aimed at delivering “individualized” to obesity patients. Many research results have been used to guide the clinical decision regarding dose adjustments of intravenous anesthetics in obese patients; besides the non-depolarizing neuromuscular-blocking agent (calculated by per-kilogram IBW), LBW is a more appropriate dosing scalar for the majority of anesthetic agents [24–26]. At present, remimazolam is administered based on TBW in the instruction manual, which is suitable for normal-weight patients, but may not be suitable for obese patients. This prospective, controlled clinical trial is designed to compare different weight-based scalars of remimazolam tosylate for anesthesia induction in obese patients.

Remimazolam is a water-soluble benzodiazepine intravenous drug, developed for the induction and maintenance of general anesthesia, short-term sedation during various diagnostic and therapeutic procedures as well as long-term sedation during ICU stay. Remimazolam combines the favorable safety profile of benzodiazepines with regard to hemodynamic stability with propofol’s fast onset and offset characteristics and improved controllability. For remimazolam induction of anesthesia, a short infusion of 6 or 12 mg/kg/h was used, and the mean time to LOC (the time when the patient became unresponsive to the shaking of their shoulder) was slightly longer for remimazolam (102 s and 88.7 s for 6 mg/kg/h and 12 mg/kg/h induction doses, respectively) when compared to propofol (78.7 s) in ASA I to II patients in the large phase IIb/III study. There were no occurrences of intraoperative arousal or recall, need for rescue sedative medication, or body movements in any patients [23]. Doi et al. also demonstrated both induction regimens (6 and 12 mg/kg/h) were equally efficacious and safe in vulnerable patients (ASA III); a significantly shorter time to LOC (81.7 s vs 97.2 s) was observed with the higher remimazolam dosage [27]. Considering that morbidly obese patients undergoing general anesthesia are at risk of hypoxemia during anesthesia induction, even after adequate preoxygenation, altered respiratory mechanics and physiology in the obese lead to an apnea time of only 2–4 min [28]. According to the previous study, the time of LOC caused by 12 mg/kg/h is closer to that of propofol, which is more suitable for rapid sequential induction strategy and reducing safe apnea time in obese patients, so in this study, we choose 12 mg/kg/h as the induction dose.

Propofol was commonly used for anesthesia induction in obese and non-obese patients; hence, there were several studies about the induction dosage selection of propofol in obese patients. By using the time to LOC (syringe drop as the marker) as the PD endpoint, Ingrande et al. proposed that LBW was the best scalar for anesthesia induction with propofol in morbidly obese (MO) subjects [26]. However, it has also been suggested that the calculation of induction dosage based on LBW is often insufficient. Subramani et al. randomized sixty MO patients to be induced with a propofol infusion of 100 mg/kg/h to a target of BIS 50 (BIS group) or until a precalculated dose of 2.6 mg/kg LBW obtained from the above-mentioned study. They found that 60% of patients in the LBW group required additional propofol that was above the predetermined dose for the MOAA/S to reach 0, which was the target anesthesia LOC endpoint [29]. The MOAA/S scale is frequently used in sedation-related drug and device studies to assess a subject’s level of sedation; the investigators delivering the trapezius squeeze will be trained using a pinch gauge (Jamar hydraulic, Patterson Medical, Boling-brook, IL, USA) to approximate this level of force. During data gathering, before the delivery of each trapezius squeeze to the subject, the investigators pinched the gauge to 10 pounds per square inch as quality control for the level of force applied. In our study, we will use MOAA/S score of 0 as the time point of an adequate LOC, to make sure a more stable hemodynamic parameter during establishing the airway, because the rise in heart rate and blood pressure may be well tolerated by healthy individuals but may have serious consequences in obese patients with their possible comorbidities of hypertension or coronary artery disease.

Induction of anesthesia in the obese population can be complicated because of the reduction of safe apneic time and the possibility of gastroesophageal reflux [30]. Relative overdosing of induction drugs predisposes to hypotension, in contrast to underdosing, which can result in the risk of awareness and hypertension. The management of obese patients who underwent Eye & ENT surgery is further complicated due to the accompanying difficult airway. Strengths of the protocol include that it is the first prospective, controlled study about the induction dose scalar and safety and efficacy of remimazolam tosylate for anesthesia management in obese patients. The results of the study will provide confidence for the application of remimazolam tosylate in obese patients undergoing Eye & ENT surgery.

## Trial status

The trial is currently at the stage of patient recruitment and data collection. The first patient was enrolled on 10 February 2022 and is expected to be finished by 31 October 2023.

## Data Availability

Any data required to support the protocol, the participant information materials, and the consent form in Chinese can be supplied from the corresponding author on reasonable request.

## Abbreviations

TBW: Total body weight
LBW: Lean body weight
BMI: Body mass index
MOAA/S: Modified Observer’s Assessment of Alertness and Sedation
PK: Pharmacokinetics
PD: Pharmacodynamics
MO: Morbidly obese
GABA: Gamma-aminobutyric acid
CSHT: Context-sensitive half-time
Vd: Volume of distribution
CO: Cardiac output
ASA: American Society of Anesthesiologists
HR: Heart rate
BP: Noninvasive blood pressure
ECG: Electrocardiogram
ETCO_2_: End-tidal carbon dioxide
SpO_2_: Pulse oxygen saturation
BIS: Bispectral index
TOF: Train of four stimulation
LOC: Loss of consciousness
IBW: Ideal body weight
EMG: Electromyogram
PACU: Post-anesthesia care unit
DMC: Data monitoring committee
ALT: Alanine aminotransferase
AST: Aspartate aminotransferase
CRFs: Case report forms
